# Oral-genital HPV infection transmission, concordance of HPV genotypes and genital lesions among spouses/ partners of patients diagnosed with HPV-related head and neck squamous cell carcinoma (HNSCC): a scoping review

**DOI:** 10.1186/s13027-023-00539-2

**Published:** 2023-10-19

**Authors:** Nadia Kalinganire, Annette Uwineza, Lynnette Kyokunda, Cecily Banura

**Affiliations:** 1 Department of Pathology, King Faisal Hospital, Kigali, Rwanda; 2 University of Rwanda, Kigali, Rwanda; 3 University Teaching Referral Hospital of Kigali, Kigali, Rwanda; 4 University of Botswana, Gaborone, Botswana; 5 Makerere University, Kampala, Uganda

**Keywords:** Human papillomavirus, Head and Neck squamous cell carcinoma, Couples/Partners/Spouses, Oral-genital

## Abstract

**Background:**

There is an increase in number of Human Papillomavirus related head and neck squamous cell carcinoma (HPV-related HNSCC) High risk HPV(HR-HPV) types can be cleared by an infected person, however, some can persist and develop HN cancer. There is a broad knowledge gap regarding HPV and related cancers.

**Main text:**

The aim of this review is to assess existing published knowledge on oral-genital HPV transmission, concordance of HPV genotypes and risk of oral or/and genital lesions among spouses/partners of patients diagnosed with HPV-related HNSCC, identify gaps in the current research and highlight areas that requires further inquiry.

**Method:**

Database like Pub med, Google Scholar, Scopus, Puplon, Wiley online library were used for search strategy. Published papers on transmission, concordance of HPV genotypes and genital lesions among spouses/partners of patients diagnosed with HPV-related HNSCC were included. Papers published from January1,2000 to October 31, 2022 were included. The published papers included are 8 Case reports, 2 cross-sectional studies, 3 Cohort studies and 2 systematic reviews.

**Results:**

A total of 2125 citations were retrieved from the five sources. 15papers were included. Case reports reported concurrent HPV-related oropharyngeal, tonsillar, unspecified HNSCC, laryngeal and nasopharyngeal carcinoma among couples. The two cross-sectional studies were done. Almost all the tumors taken from patients with HPV-related oropharyngeal carcinoma (HPV-related OPC) and their spouses were positive for identical HPV 16 type. The three cohort studies showed an increase risk of upper aero-digestive tract cancer among male spouses of females with cervical cancer. Two systematic reviews reviewed literature studies which evaluated concurrent cases of HPV-related Oropharyngeal cancers. Examination of these papers showed that the majority of the studies suggested that there is HPV transmission, concordance and risk of HNSCC cancer among spouses with HPV-related oral-genital cancer. No studies evaluated the risk of developing genital cancer in spouses of patients with HNSCC.

**Conclusion:**

The findings of this review highlighted big need of further research on oral-genital HPV infection among spouses of patients diagnosed with HPV-related HNSCC. Studies are needed to evaluate the risk of getting genital and upper aero-digestive tract HPV-related cancer among spouses with HPV-related HNC.

## Introduction

Head and neck cancers (HNCs) is a heterogeneous group of cancers which includes cancer of the oral cavity, pharynx, and larynx, and is the seventh most common cancer worldwide with an estimated annual incidence of over 650,000 and mortality of 300,000 [1, 2].

Recent studies show that the incidence ofHPV-related HNC in rapidly increasing particularly in high income countries [3, 4]. The increase is more evident among males in high income countries compared to those in developing African countries. Generational differences in sexual behavior or increased persistence or progression of oral HPV due to changes in co-factors may explain the observed increase [5].

The etiological role of HR-HPV in development of HNCs has been well established in recent studies. It is almost certain that oropharyngeal HPV infection necessary in the development of HPV-related HNCs [6, 7].

It is estimated that about 25% of HNC- squamous cell carcinomas (HNCSCC) are caused by HR-HPVs [8]. HPV types associated with HNC are transmitted primarily through oral sex defined as contact of the oral region with the anogenital region such as vagina, anus, penis and in general, the external genitalia. Oral sex is practiced by males more than females. The risk increases with an increase in the number of oral sexual partners [9–11].

An international cross-sectional study done by Castelleague et al. on 3680 tissue biopsy specimens has shown HPV- Deoxyribonucleic Acid (HPV-DNA) prevalence of 24,9%, 21,4%, 7,9%, 7,4%, 7% and 3,9% in oropharynx, unspecified pharynx, nasopharynx, oral cavity, larynx, and hypopharynx respectively [12].

High-risk type 16 constitutes the most prevalent HRHPV type globally, being detected in almost 60–80% of head-and-neck cancers. However, high-risk type 18 was found in 34% of oral cavity squamous cell cancers and 17% of laryngeal squamous cell cancers [8, 13].

It is important to note that infections with HPV 16 and HPV 18 infections also cause almost all cases of cervical cancer, a percentage of other anogenital cancers (vaginal, vulva, anal, penile) [14].

Recent studies seem to suggest that prophylactic HPV vaccines primarily tested and registered for use against genital HPV infections have a strong protective effect against oral HPV 16 and HPV 18 infections, which contribute to the vast majority of HPV-positive head and neck cancer cases worldwide [15]. Studies show comparably high efficacy of HPV vaccines in preventing oral HPV, which arises from vaccine-induced robust systemic neutralizing antibodies, the likely effector mechanism against mucosal HPV infections [16]. Indeed, recent studies have demonstrated a direct benefit of prophylactic HPV vaccination among young adults (ages 18–33 years), where prevalence of vaccine-type oral HPV infections (HPV16/18/6/11) was 88% lower in men and women who self-reported receipt of at least one dose of the HPV vaccine, including a 100% reduction in vaccinated men [17]. Further, emerging evidence also suggests considerable indirect benefit through herd protection against vaccine-type oral HPV infections. In one study, vaccine-type (HPV16/18/6/11) oral HPV prevalence declined by 37% during 2009–2016 in unvaccinated US men aged 18–59 years, without a commensurate change in the prevalence of non-vaccine oral HPV infections. These results suggest herd protection among unvaccinated men arising from increased vaccine uptake in females [18].

Oral and genital HPV transmission and concordance of HPV types in couple has been reported by different authors. Studies showed that women previously diagnosed with cervical/vaginal HPV infection or high grade cervical intraepithelial neoplasm(CIN), are significant reservoir for HPV infection; and their male partners are at risk of oral HPV [19–21]. A narrative review by Malgorzata notified that oral-genital transmission was proven to be the best documented route for oral HPV infection, depending on whether there is oral sex practice, presence of a local HPV infection in one of the partners and having oral contact with a partner with oropharyngeal cancer(OPC) [21]. Concordant HPV in oral samples male partners of women with CIN had been stated by different studies [19, 22–24]. Lea et al. has shown that certain hygienic and sexual behaviors are associated with anogenital concordance between healthy monogamous heterosexual couples [25]. Other concordance were found in published case reports on concurrence HPV-related oropharyngeal, nasopharyngeal and tonsil SCC, where both partners were found with the same HPV types in their tumors [26–28]. Also there was concordance between OPC in husbands of wives previously diagnosed with cervical cancer [29, 30]. However, another studies showed that asymptomatic HPV infection was common in both partners but the concordance was low [31–36].

There is limited published literature about the epidemiology and natural history of HPV-induced HNCs. For instance, current knowledge about potential transmission of HPV infection between couples or intimate partners/spouses of patients with HNCs are based mainly on few case reports, which are inconclusive. Further, concordance of HPV types or HPV genomes between couples or intimate partners reported in the few published case reports does not rule out co-infection from an exposure to third person/persons having this same virus. A systematic review done by Sean et al. on HPV knowledge among patients and health care providers has shown a knowledge gap of HPV-related oropharyngeal squamous cell carcinoma(OPSCC) for both the general population and Health care providers [37]. Studies done in Africa about HPV knowledge have shown limited knowledge among respondent [37]. A systematic review by Cunningham et al. highlighted a broad knowledge gap regarding HPV and cervical cancer [38]. Although there are almost no studies done in developing countries on HPV infection transmission and concordance among spouses, the delay in doing cancer screening and diagnosis can reinforce the increase of HPV-related cancers. Therefore, strategies to improve awareness on HPV infection among spouses as well as HPV-related cancers is needed for prevention.

There is no evidence to show whether spouses or intimate partners of patients with HPV-related HNC may be at increased risk of developing an HPV-related lesion or malignancy in oral and/or genital regions. Yet, this information is important to develop interventions for spouses or partners of patients with HNCs [35]. Therefore, the objectives of this scoping review are to assess the knowledge of published articles on (i) Oral-genital HPV transmission and concordance of HPV genotypes and genital lesions among spouses/partners of patients diagnosed with HPV-related HNSC (ii) Risk of oral and/ or-genital lesions in spouses of patients with HPV-related HNSCC and (iii) identify gaps in the current research and highlight areas that requires further inquiry.

## Methodology

### Protocol

This scoping review adhered to Preferred Reporting Items for Systematic reviews and Meta-Analyses extension for Scoping Reviews (PRISMA-ScR) Checklist [39].

### Eligibility criteria

#### Inclusion criteria

- Papers on oral- genital transmission, concordance of HPV infection among couples or sexual partners/spouses of patients with HPV-related HNSCC.
- Published from January1, 2000 to October 31, 2022.
- Restricted to only English language publications.

#### Exclusion criteria

- Papers published as letters, in books and in grey literature.

### Information source and search strategy

One author conducted an initial search relating to the review topic in order to identify key words or phrases found in titles or abstracts to inform the design of the search strategy. Next, the same author designed a comprehensive search strategy and formally searched the selected electronic databases including Pub Med, Scopus, Google scholar and Wiley outline library.

The following key terms were used for the search strategy of all databases: carcinoma/squamous cell/head and neck neoplasm/oropharyngeal neoplasm/mouth neoplasms/squamous cell of head and neck” AND Papillomaviridie/papiloma infections/human papillomavirus/HPV positive/p16positive) OR (“oropharynx cancer/oropharyngeal carcinoma/opscc/oropharynx tumor” AND human papillomavirus/HPV/HPV positive/p16 positive”) OR (“larynx cancer/oropharyngeal carcinoma/larynx tumor/mandibular cancer/tonsillar cancer/Nasopharyngeal cancer, nasal sinus.

Additional titles were obtained from reference lists of the initial search, which were published within the review time restriction, full text papers included in the review. Reference lists of related reviews were also scanned to identify additional titles.

### Article selection

During the initial screening (level 1), one author screened citations (titles and abstracts) to identify and remove duplicates. Then, two authors independently applied the eligibility criteria to consider potential titles for inclusion during level 2 screening. Disparities in title selection were discussed and resolved in order to build a full article list. Full articles of titles that fulfilled the inclusion criteria were retrieved and reviewed.

### Data abstraction, items and charting

Two authors were involved in data abstraction. The first author abstracted data from the selected articles using PRISMA-ScR guidance and the second validated the abstracted data. Study characteristics i.e. first author, year of publication, country where study was conducted, objectives, study design, study population and main findings were abstracted from each selected article.

### Methodological quality appraisal

The authors did not check for methodological quality or risk bias of the selected papers, which is consistent with the scoping review conduct [40].

### Data presentation and synthesis

The abstracted data were presented in a chart (Table 1). Data synthesis included frequency analysis of country where the study was conducted.

### Targeted audience

The primary audience of this review include but not limited to Public health professionals, ENT clinicians, Gynecologists and pathologists.

## Results

**Fig. 1:**
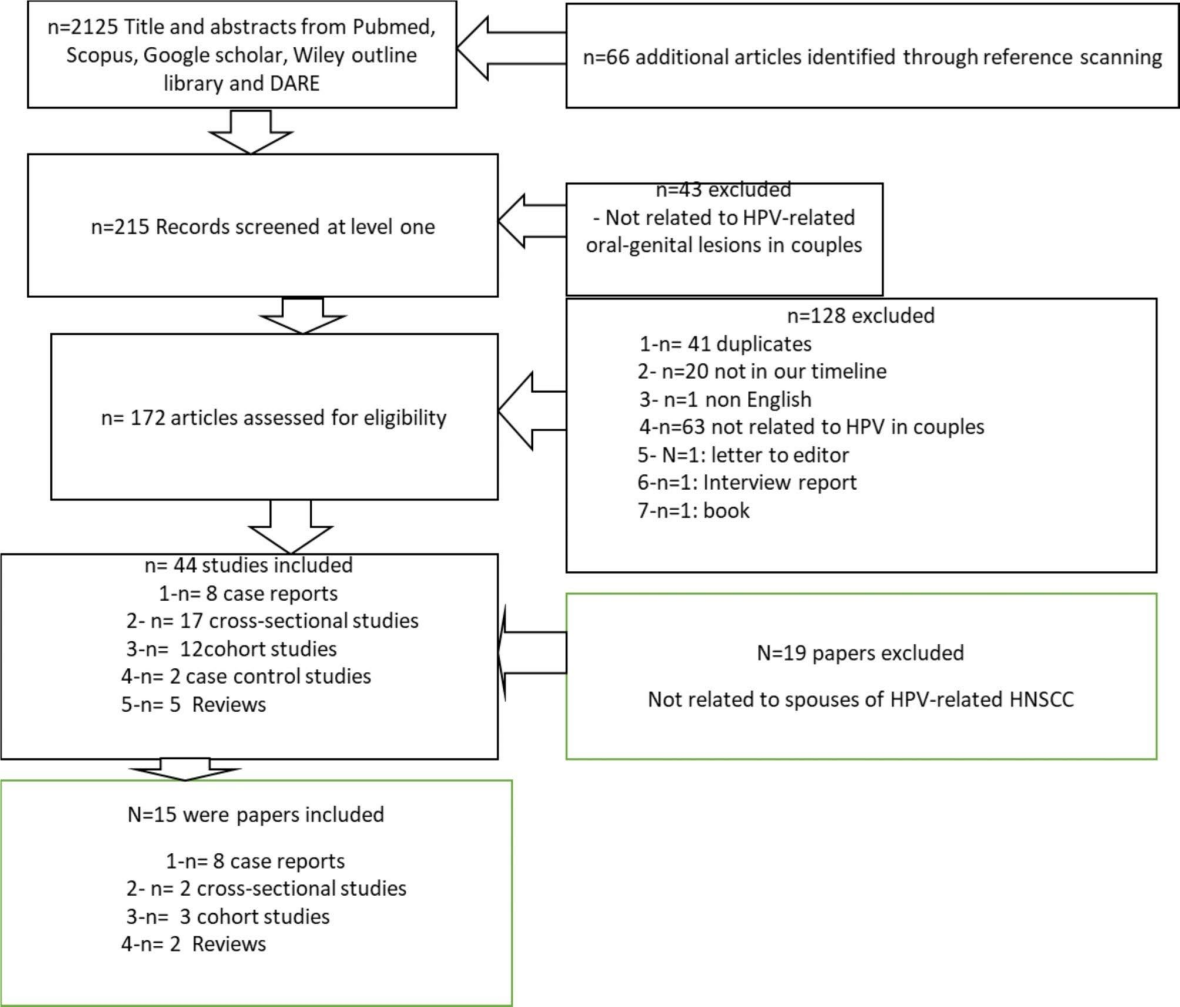
Study flow: Details of the flow information through different phases, number of records identified, included and excluded and reasons for their exclusion

A total of 2125 citations were retrieved from all sources. After screening 215 potentially relevant papers at level 1, 43were excluded for not to be related to HPV –associated oral-genital lesions in couples. of HPV-related HNSCC patients. At level 2,172 articles and abstracts were assessed for eligibility. 128 articles were excluded: 41 duplicates, 20 were not in our timeline, 1 non English, 63 were not related to HPV in couples, 1 letter to editor, 1 interview report and 1 book. From 44 studies included, 19 were excluded for not related to spouses of patients of HPV-related HNSCC. Eight duplicates were excluded, and 1article was excluded for being inaccessible. 13 papers and 2 abstracts were included in the review. Figure 1. Among these 15 articles were: case reports(n = 8), cross-sectional studies(n = 2), retrospective cohort studies(n = 3) and reviews(n = 2) The studies were conducted in: North America: USA (n = 6); Asia: Taiwan(n = 1), India(n = 1), Japan(n = 1); Europe: Germany(n = 1), France(n = 1), Sweden(n = 3), UK(N = 1).

**Table 1:** Data extraction characteristics and findings

First Author, year of publication and location	Study design	Study population	Aims	Main Findings
1. Isao Vemaetomari, 2007, Japan [41]	Case report	One married couple	Report a married couple with tonsillar SCC	Same HPV-16 subtype found in tonsillar SCC
2. Robert Haddad, 2008, USA [42]	Case report	One couple, husband and wife	Report a couple, husband and wife diagnosed synchronously with head and neck squamous cell carcinoma	HPV-16 positive by polymerase chain reaction(PCR) sequencing and identical genomes, which were closely related to the revised European prototype, HPV-16R
3. Elizabeth Andrews, 2009, USA [28]	Case report	2 couples	Describes 2 non- smoking couples who developed HPV-related tonsillar cancer within 12 months of each other	Identical DNA sequences of HPV-16 L1, LCR, and E6/E7 regions
4. Lee-Wen Huang, 2010, Taiwan [29]	Case report	2 couples	Report 2 patients with HPV-associated cervical cancer and synchronous diagnoses of HPV-related HNC of their husbands.	Couple 1 HPV-16 DNA detected in cervical cancer and in laryngeal cancer tumor specimen of the male spouse using PCR. Couple 2 HPV-31 DNA detected in female spouse tumor specimen. HPV-18 detected in nasopharyngeal carcinoma of male spouse
5.Tyler D. Brobst, 2016, USA [27]	Case report	1 married couple	Describe the case of a married couple who presented with HPV-positive Oropharyngeal carcinoma within two months each other	Both tumors were HPV-16 and P16 positive. Nearly identical HPV-16 genomes by Sanger sequencing
6. Brobst. Daniel, 2017, USA [26]	Case report	1 couple	To report on unusual case of HPV-related Nasopharyngeal cancer occurring in a male patient whose wife had cervical cancer	HPV type 16 positive and P16 positive nasopharyngeal carcinoma in male spouse of partners with cervical cancer positive for P16 but unknown HPV type
7. Hans Prakash Sathasivam, 2018,UK [43]	Case report	4 heterosexual couples	Report the clinic pathological features of a further four couples with HPV-related Oropharyngeal carcinoma and compare them with published cases	HPV-16 and P16 positive. HPV-related oropharyngeal squamous cell carcinoma
8 .J.M. Vahl, 2018, Germany [44]	Case report	1 married couple	Report a metachroneous oropharyngeal carcinoma in a married couple	HPV positive tumors
9. Gypsyamber D’Souza, 2014, USA [45]	Multicenter prospective studies	164 patients with HPV-related oropharyngeal carcinoma and 93 of their spouses	To better understand oral HPV infection and cancer risk among long-term sexual partners with HPV-positive Oropharyngeal cancer	Unconfirmed reported HPV-related cancer history. Several patients reported a previous partner who had been diagnosed with an HPV-related cancer including invasive cervical cancer(n = 3;2.0%), oropharyngeal cancer(n = 2; 1.4%) and anal cancer(n = 1;0.7%).
10. Anne S.Tsao, 2016, USA [23]	Cross-sectional	227 patients partners pairs enrolled	To evaluate the prevalence of oral HPV and assess which risk factors may be associated with increased transmission rates of patients with oropharyngeal carcinoma and their partners	Of 144 patients with oropharyngeal tumor tissue, 128(89%) had HPV positive tumors. Oral HPV-16 more frequently detected among patients and partners (9% and 6%, respectively). Concordance between the oral HPV status of the paired patients and partners. HPV detected in oral swabs of 19 couples HPV-16: 11(58%) couples HPV 56: 8(42.1%) (4 in conjunction with HPV-16). Multiple HPV genotypes:9 positives for multiple HPV genotypes
11. K. Hemminki, 2000,Sweden [30]	Prospective cohort study	2740 women diagnosed with cervical cancer retrieved from nationwide Swedish family cancer database	To probe the possible role of HPV infection in squamous cell carcinoma of the upper aero digestive tract, with a special reference to tonsillar cancer as well as the first cancer of their husbands.	An excess standardized incidence ratio (SIR over 2.00) of both tonsillar cancer(SIR 2.39) when wife with in situ cancer and SIR 2.72 when wife with invasive cervical cancer and cancer of the tongue
12.Weires Marianne, 2011, Sweden [46]	Retrospective cohort-linkage study	3.5 million families and 16 million individuals in the population based Swedish family data linked to cancer diagnoses	To quantify the contribution of spousal environment sharing to a cancer risk and to explore the clustering of cancer types among spouses	Increase risk of upper aero digestive tract among male spouses of females with cervical cancer (SIR 1.41). Increase risk of cervical cancer among female spouses with upper aero digestive tract (SIR 1.33). Increase risk of concordance among spouses with aero digestive tract cancer
13.Tuomas Lehtinen, 2022, Sweden [47]	Retrospective cohort study	3.5 million families and 16 million individuals in the time period of 1969–2001 and 2002–2015	To evaluate relative risk of tongue squamous cell carcinoma and base of tongue squamous cell carcinoma in the husbands of women with ano- genital HPV-associated cancer using Swedish family data linked to cancer diagnosis	Increased relative risk of tongue and base of tongue cancer among male spouses of females with in-situ cervical cancer both in the time period of 1969–2001 and 2002–2015. Increased relative risk of tongue squamous cell carcinoma/ base of tongue squamous cell carcinoma in male spouses of females with invasive ano- genital cancer Peak relative risk of 9.4 in 2002–2015 in spouses diagnosed with invasive cancer both at age less than 50 years.
14. Haitham Mirghani, 2017, France [35]	Systematic review	53 publications in the time period of 1933 to 2016	To perform a systematic review of the literature studies evaluating concurrent cases of HPV-induced oropharyngeal cancers in partners of patients with oropharyngeal or cervical cancer	Concurrent tonsil cancers in four couples from which the same HR-HPV strains were involved in husbands and wives. Two couples with concurrent HPV-related nasopharyngeal and cervical cancer. Matching strain was not performed. Relative risk of tongue and tonsil cancer in husbands of women diagnosed with cervical cancer was 2.42 and 2.72 respectively.
15. Prashanth Panta, 2019, India [48]	Review	5 publications	Review published studies in couples with concurrent OPSCC	Concurrent OPSCC were more often seen at advanced age

### Oral-genital HPV transmission and concordance among spouses/partners of patients diagnosed with HPV-related HNSCC

Several case reports about concurrent HPV-related Head and Neck squamous cell carcinoma have been published which seems to suggest a possibility of direct horizontal HPV transmission between intimate couples. Isao et al. reported a married couple with the same HPV type detected in their tonsillar Squamous cell carcinoma tissue specimens [41]. Haddad et al. reported a couple with HNSCC in which both tumors positive for HPV 16 were found to have identical HPV genomes [42]. Andrew et al. described 2 non-smoking couples with HPV 16 -related tonsillar cancer were found to have identical DNA sequences of the HPV-16 genome in L1, LCR, and E6/E7 regions [28]. Huang et al. reported 2 couples with HPV-related cancers. In one couple, HPV-16 was detected in cervical SCC of the wife and HPV 16 was detected in laryngeal carcinoma tissue specimens of her husband. For the second couple, however, HPV 31 was detected in the cervical tumor and HPV 18 in nasopharyngeal tumor from the husband [29]. Brobst et al. reported a married couple both diagnosed with HPV-related oropharyngeal carcinoma and nearly identical the HPV strains detected in tumor specimens [27]. Vanderbilt et al. reported a male spouse with HPV 16 detected in nasopharyngeal cancer whose wife had cervical cancer of unknown HPV type [26]. Prakash et al. reported four heterosexual couples with HPV16-detected in their oropharyngeal SCC [43]. Vahl et al. reported a married couple with HPV positive metachroneous oropharyngeal SCC [44]. The review by Panta et al. has shown that the concurrent OPSCC were more often seen in advanced age. Almost all the reported cases showed concordant HPV type which shows a possible HPV transmission in couples [48]. More studies/reports on concurrent HPV-related HNSCC in couples and the matching of the strains should be performed from every case to improve understanding of HPV infections among spouse/ sexual partners of patients with HPV-related HNSCC.

### Risk of oral and/ or-genital lesions in spouses of patients with HPV-related HNSCC

To better understand cancer risk among long-term sexual partners with HPV-positive oropharyngeal cancer, D’Souza et al. conducted a cross-sectional study among 164 patients with HPV-related oropharyngeal cancer and their spouses The study findings show that several patients reported a previous partner who had been diagnosed with an HPV-related cancer, including invasive cervical cancer (n = 3; 2.0%), oropharyngeal cancer (n = 2; 1.4%), and anal cancer (n = 1; 0.7%). In addition, one partner with current spouse patient with HPV-related OPC reported a previous husband who died as a result of oropharyngeal cancer [45]. However, these findings could not be confirmed because previous partners were not studied. Hemminki et al., conducted a retrospective cohort study using records of Swedish family cancer database The findings show an excess (SIR over 2.00) of both tonsillar cancer (SIR 2.39) when wife had in-situ cervical cancer and SIR 2.72 when wife had invasive cervical cancer and cancer of the tongue [30]. Tuomas at al in a prospective cohort study linked 3.5 million families and 16 million individual’s Swedish family data to cancer diagnosis. The findings show increased relative risk (RR) of tongue and base of tongue cancer among male spouses of females with in-situ cervical cancer both in the time period of 1969–2001 and 2002–2015. In addition, increased RR of tongue squamous cell carcinoma/Base of tongue squamous cell carcinoma in male spouses of females with invasive anogenital cancer. The peak RR is 9.4 in 2002–2015 in spouses diagnosed with invasive cancer both at age less than 50 years [47].

Weires et al. also showed that husbands of women with cervical cancer had an increased risk of upper aerodigestive tract with a SIR 1.41 and wives were at risk of developing cervical cancer when their husbands had upper aerodigestive tract cancer, SI R 1.33 [46]. Although concurrent tumors were found among spouses, HPV status in tumors was not studies. There is still lack of evidence that these cancers were HPV- related.

### Genital HPV infection and lesions in spouses of patients diagnosed with HPV-related HNSCC

We found no studies done evaluating cervical HPV infection/lesion in female spouses of patients with HPV-related HNSCC as well as genital HPV infection/ lesions in their male partners. Although it is still not clear whether spouses of patients with HPV-related HNSCC can get genital infections and/or lesions, some authors, in their propositions, they stated that women of patients with HPV-related HNSCC should be screened regularly for cervical cancer [45, 49]. All published studied seem to be among heterosexual couples. GAP that needs further research: Studies among homosexual males, who in many countries are not eligible to receive preventive HPV vaccines.

## Discussion

The objective of this review are to identify gaps in the existing knowledge regarding oral-genital HPV infection transmission, concordance of HPV types and genital lesions among spouses/partner of patients diagnosed with HPV-related HNSCC, and suggest future research directions.

We found that most published studies were mainly case reports conducted in North America and Europe. No studies have been conducted or published from the African continent.

Literature on oral-genital HPV transmission and concordance of HPV types is still inconclusive. While some papers reports high, others report low transmission rate [45, 50, 51].

HPV transmission in couples without cancer has been previously reported including oral- oral, oral-genital as well as genital-genital transmission. Studies have reported a higher rate of HPV transmission from the female ano-genital tract to the males than from males to females probably because there is liberation on oral sex practice [34]. The nature of epithelial cells of penis seem to be more resistant to HPV infection than cervical epithelial cells. In addition, the duration of HPV infection is shorter in men than in women [51]. It is also possible that the low level of oral-genital HPV transmission is probably due to the fact that the majority of people are able to clear oral HPV infection [45]. It is also possible that there may be more homosexual couples than reported in the literature, which probably reduced the rate of oral-genital transmission in couples [45]. Further, the sampling technique using a brush particularly in tonsillar crypts may not collect the intended sample resulting in false negative oral-genital HPV transmission result [52]. The low oral HPV transmission has been reported in couples that performed oral-genital sex or had a high number of open-mouthed kissing than couples who performed genital-genital sex [9]. These findings support the findings that males practicing oral sex and being a male partner of a woman with CIN lesion are risk factors associated with HPV transmission in couples [34, 53].

Published literature seem to suggest that HPV transmission by oral-genital sex or by self-inoculation among sexual partners to the oropharynx is a rare and unlikely event with low concordance of HPV types. This was explained by the fact that low oral prevalence could be due to an independent clearance of HPV from the oropharyngeal compared to the cervix. This is similar to the study done by by Elizia et al. which suggested that the presence of cervical lesion does not lead to HPV oropharyngeal infection. This study also brought out the low-rate of HPV infection in the oropharyngeal mucosa of women with cervical lesions and their partners [50]. Gianguido et al. reported that there is a risk of subclinical oral HPV infection in women with cervical HPV and their male partners [19].

The risk of OPSCC in men has been reported to be higher when their female partners have HPV-related cervical intraepithelial neoplasm [34].

Literature on transmission and concordance is still inconclusive. While some papers reports high, others report low [45, 50, 51]. D’Souza et al. suggested that it was due to HPV clearance. This is consistent with suggestions from other studies that the majority of people are able to clear oral HPV infection [45]. Another reason given in the study by D’soza et al. was that there were more homosexual couples which probably reduced the rate of oral-genital transmission in couples [45]. Other suggested possible causes of the low prevalence of HPV infection from a different study is that, due to that HPV infection is known to be localized in tonsillar crypts, HPV DNA might not be reached by the brush during sample collection [52]. The low transmission was mostly seen in couples which depend upon the type of sexual relationship. D’souza reported that couples performing oral-genital sex or a high number of open-mouthed kissing are more likely to have oral HPV transmission than those who perform genital sex [9]. These findings support the suggestions that practicing oral sex as it was reported as main transmission route, being a male partner of a woman with CIN lesion and being a female transmitting the infection are factors associated with HPV transmission in couples [34, 53]. Ralf et al. suggested that a higher rate of transmission was observed from the female ano-genital tract to the male genitals that the other way round [34]. Two studies done in Spain suggested that the persistence of high risk HPV-related lesion in one partner may cause clearance more difficult to achieve in another [53]. This statement is supported by Diez et al. who reported that the proportion of female with the same genotype as their male partners was higher that the proportion of male sharing the same genotypes as their female partners.

Studies have addressed the issue of cancer risk in couples. The risk of HNSCC in partners of patients with cervical cancer was assessed in a retrospective data collected in cancer registry in Sweden. In this study, Hemminki et al. has have shown that husbands of women with cervical carcinoma in situ and women with cervical cancer were diagnosed with tonsilar cancers with SIR of 2,39 and SIR of 2,72, respectively [30]. The limitation in these findings is that there was no evaluation whether these HNSCC were associated with HPV and also the HPV concordance in these couples. Weives reported a relatively lower SIR of 1,41 risk of upper aero-digestive tract cancer among spouses of females with cervical cancer [46]. Tuomas et al. also suggested the same findings reporting that relative risk for TSCC/BOTSCC increased by time and reduced by age in two different period [47]. Haitham et al. reported a relative risk of tongue and tonsil cancer in husband of women diagnosed with cervical cancer of 2,42 and 2,72 respectively [35]. All these suggestions support the idea that husbands of women with CIN are more likely to have oral HPV infection. Case reports have also been published about couples from which husbands who’s wives were diagnosed with cervical cancer laryngeal and nasopharyngeal SCC [26, 29].

Concordance of HPV types in couples has been reported in several case reports. However, it seems that the proportion of female with the same genotype as their male partners was higher than the proportion of male sharing the same genotypes as their female partners probably because the duration of HPV infection is shorter in men than in women [51].

Non-concordance (different simultaneous) of HPV types in couples has also been reported. In one case report, HPV type 31 was detected in sample of a wife with HPV type 18 detected in husband’s sample(Huang). It is possible that couples could have different HPV types due to that co-infection in couples is no longer expressed after several years. Although the reviewed papers in this review did not examine when individuals usually exposed to the virus and the timeframe of cancer HPV-related HNSCC development in couples. However, for partners in long standing relationships for at least a decade, it’s probably enough to facilitate HPV driven carcinogenesis. And also, advanced age can reduce the ability to combat the virus. Astudy by Sathasivan et al. on concurrent HPV-related OPC in four couples showed the mean age of 63 years. The mean duration of relationships was 36 years and the interval between the index cancer and partner’s cancer varied between 16 and 64 months [43]. The case reports reviewed reported couples whose partner’s age were more than 50 years with long standing relationships varying between 15 and 31 years of marriage [26–29]. Huang et al. reported a couple of a 64 years old woman with cervical cancer and a husband with laryngeal cancer. This couple had been practicing oral genital sex for 20 years [29]. However, Haddad reported a couple with the same age of 75 years and suggested that HPV 16 infection occurred and was transmitted within a short time frame. This statement was based on the subsequent appearance of the tumor [42]. Therefore, it is possible that both partners may carry HPV infection before their meeting which might explain similar time course of cancer development.

Concurrent cases of HPV-related oropharyngeal carcinoma and tonsillar carcinoma have been reported. Prakash et al. described four heterosexual couples with concurrent HPV 16-related oropharyngeal carcinoma [43]. Brobst also reported described a case of a married couple who presented with HPV-positive oropharyngeal carcinoma within two months each other [27]. Haddad et al. described a case report of husband and wife who were diagnosed synchronously with HNSCC with tumors positive for HPV16 [42]. Andrew et al. described a case report of two non -smoking, non- drinking couples who developed HPV-associated tonsillar cancer within 12 months of each other [28]. The number of published papers related to the risk of oral-genital cancers in couples used in this scoping review is 7 which relatively higher than 3 that used in a systematic review by Mirghani et al. This mean that, although there are still few literatures on this subject, the fact that cases are under reported cannot be ignored. None of these studies reported on evaluation genital lesions from spouses of patients diagnosed with HPV-related HNSCC.

A couple of strength and limitations in this review should be considered: The strength of this review depends on our systematic search of the databases and independent screening by two reviewers. We did not search for grey literature, leaving the review susceptible to publication bias. The extent of this bias and study quality were not assessed as per scoping review recommendation. One of the limitations was that the majority of studies were case reports; which are the weakest in hierarchy of research evidence. Another limitation is that the search of study papers was limited to those available electronically and this could have affected the retrieval and achievement of relevant information.

In conclusion, generally, our review highlights a considerable need of studies evaluating the risk of HPV-related cancer in spouses of patients with HPV-related HNSCC. Studies are needed to evaluate the risk of getting genital HPV-related cancer among spouses with HPV-related HNSCC. Studies are needed in both heterosexual and homosexual couples.

## Data Availability

All data generated during this review are included in this published article and can be available from the corresponding author on a justified request.
